# Avenanthramide-C prevents noise- and drug-induced hearing loss while protecting auditory hair cells from oxidative stress

**DOI:** 10.1038/s41420-019-0195-1

**Published:** 2019-07-08

**Authors:** Alphonse Umugire, Sungsu Lee, Dami Kim, Munyoung Choi, Hyung-Seok Kim, Hyong-Ho Cho

**Affiliations:** 1 Department of Otolaryngology-Head and Neck Surgery, Chonnam National University Medical School and Chonnam National University Hospital, Gwangju, Korea; 2 Department of Biomedical Science, College of Medicine, Chonnam National University Graduate School, BK21 PLUS Center for Creative Biomedical Scientists at Chonnam National University, Gwangju, Korea; 3 Department of Forensic Medicine, Chonnam National University Medical School, Gwangju, Korea

**Keywords:** Drug development, Cell death in the nervous system

## Abstract

Noise exposure or ototoxic drugs instigate various types of damage to the cochlea, resulting in hearing loss (HL). While the incidence of HL is growing continuously, there are, so far, no adequate drugs to prevent or treat HL. Avenanthramide (AVN), a natural product extracted from oats, has been reported to possess anti-oxidant/inflammatory properties, and protect several types of cells. In this study, we investigated whether AVN-C can protect auditory hair cells, and preserve hearing from noise trauma and ototoxic drugs. Wild-type C57BL/6 mice were used to generate several HL models. Serum and perilymphatic fluid samples were analyzed using mass spectrophotometry to detect AVN-C. AVN-C crossed the blood-labyrinth barrier, and was detected in the perilymph after systemic injection. Pretreatment by AVN-C 24 h before exposure to temporary threshold shift noise contributed to the preserving hearing. Moreover, in the case of permanent threshold shift, AVN-C provided significant protection from noise. AVN-C also strongly protected against deterioration in hearing due to kanamycin and furosemide (K + F). According to the results of our scanning electron microscopy analysis, many outer hair cells (OHCs) were destroyed by noise trauma, while AVN-C prevented these losses. OHC loss due to K + F was even more severe, even affecting the apex. Strikingly, AVN-C treatment maintained OHCs at a level comparable to normal cochlea. AVN-C reduced the dichlorofluorescin (DCF)-positive population in gentamicin-treated HEI-OC1 in vitro. The expressions of TNF-a, BAK, IL-1b, and Bcl-2 were attenuated by AVN-C, revealing its antioxidant effects. The results of this study show that AVN-C crosses the blood-labyrinth barrier and provide a significant protection against noise- and drug-induced ototoxicity. Hence, AVN-C is a good candidate for future therapy aimed at protecting against sensorineural HL.

## Introduction

Hearing loss (HL) is the most common sensory disorder, affecting ~6.8% of the global population, and was ranked as the fourth leading cause of “years lived with disability” in 2013 and 2015, following lower back and neck pain, depressive disorder, and iron-deficiency anemia^1^. Sensorineural hearing loss (SNHL), damage to the inner ear or auditory neural pathways, is especially problematic, because, as yet, there is no definitive treatment, despite many studies related to drug development^2^.

There is strong evidence that oxidative free radical formation is a crucial pathway for SNHL. Noise-induced hearing loss (NIHL) is the most well studied of the deficits in the SNHL category. Exposure of C57BL/6J mice to 110 dB SPL (decibel sound pressure level) noise for 1 h led to a fourfold increase in hydroxyl radicals in the perilymph, along with outer hair cell (OHC) damage^3^. Several cascades were suggested to be involved in the development of reactive oxygen species (ROS) leading to hair cell death^4^. Broadband 120 dB SPL noise for 3 h per day for 2 days (d) to C57/129/cv mice increased iNOS and NO expression in the stria vascularis^5^, showing that the stria vascularis is also involved in noise and ROS production. Drug-induced ototoxicity is also associated with ROS. GM (gentamicin) transtympanic injection into guinea pigs elevated NO synthase II and xanthine oxidase^6^. The ototoxic effects of GM were reduced by NO synthase inhibitors or O_2_ and peroxynitrite scavengers. Cisplatin, a well-known anticancer drug causing drug-induced hearing loss (DIHL), has been reported to be related to ROS, and several scavenger agents can rescue hair cells from damage^7,8^. In the case of age-related HL, caloric restriction reduces oxidation by activating Sirt3 and increasing NADPH levels^9^. Hence, antioxidant therapy has been proposed as a preventive treatment for SNHL.

Avenanthramide (AVN) is a phenolic compound with a low molecular weight of ~300 g/mol^10^, and was originally extracted from oat grain (Avena sativa L.)^11^. Polyphenol is the most abundant compound with antioxidant effects found in the diet^12^. There are several forms of AVN in oats, of which AVN-C is the most prevalent and has the highest antioxidant activity^11^. When AVN was used to treat human aortic endothelial cells, it reduced proinflammatory cytokines and adhesive molecules, demonstrating its antiatherosclerosis effects^13^. Also, in a similar model, AVN had an antiproliferative effect on vascular smooth muscle cells, and increased NO production, leading to the prevention of atherosclerosis^14^.

In this study, we show that AVN-C provides a strong protective effect on ototoxicity using the in vivo NIHL and DIHL models, as well as ex vivo HEI-OC1 cells. AVN-C, when administrated systemically, crosses the blood-labyrinth barrier, and can act directly inside the cochlea. As it is derived from oats, AVN-C can be consumed as a dietary product. Hence, it may offer a convenient and effective drug for preventing SNHL.

## Results

### AVN-C resolves transient hearing impairment induced by noise trauma

**Fig. 1:**
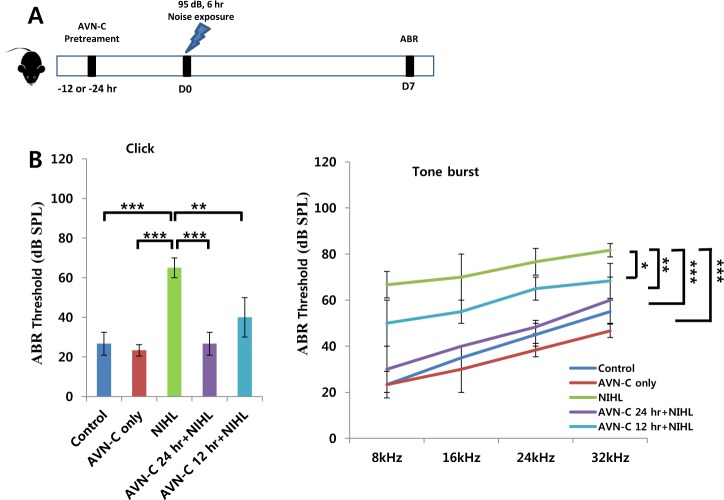
Hearing test for AVN-C treatment in the transient threshold shift (TTS) noise-induced hearing loss model. **a** Experimental timeline. AVN-C was provided intraperitoneally to the control models (without noise) and either (12 or 24) hours prior to exposure to a single noise trauma with 95 dB SPL. A week later, the auditory brainstem response (ABR) was evaluated using click and tone burst stimuli. **b** AVN-C effectively prevented transient hearing loss due to noise. *n* = 3 per each group, *P < 0.05, **P < 0.01, ***P < 0.001

We first evaluated the in vivo effects of AVN-C on hearing using temporary threshold shift (TTS) noise trauma models. A single pure tone 95 dB sound at a frequency of 8 kHz was played for 6 h to induce a TTS. AVN-C (10 mg/kg body weight) was delivered by intraperitoneal (IP) injection to controls (without noise) and either 24 or 12 h prior to the noise. A week after the noise, hearing levels were evaluated using an auditory brainstem response (ABR) test. AVN-C itself did not affect hearing in the control group, which was not subjected to noise trauma. The hearing threshold was elevated to 65.0 ± 5.0 dB SPL in the noise only group, while it was 26.6 ± 5.7 dB SPL in the control group (Fig. 1). Systemic AVN-C 24 h prior to noise markedly rescued the TTS induced by the noise (26.6 ± 5.7 dB SPL, *P* < 0.001, compared with the noise only group). Pretreatment with AVN-C 12 h prior to noise exposure prevented hearing damage, as indicated by the threshold of 40.0 ± 10.0 dB SPL (*P* < 0.01 compared with the noise only group), which shows that both time points are effective at preventing TTS (Fig. 1b). Hearing thresholds for all groups were normalized after a month (data not shown).

### AVN-C prevents permanent hearing loss caused by noise trauma

**Fig. 2:**
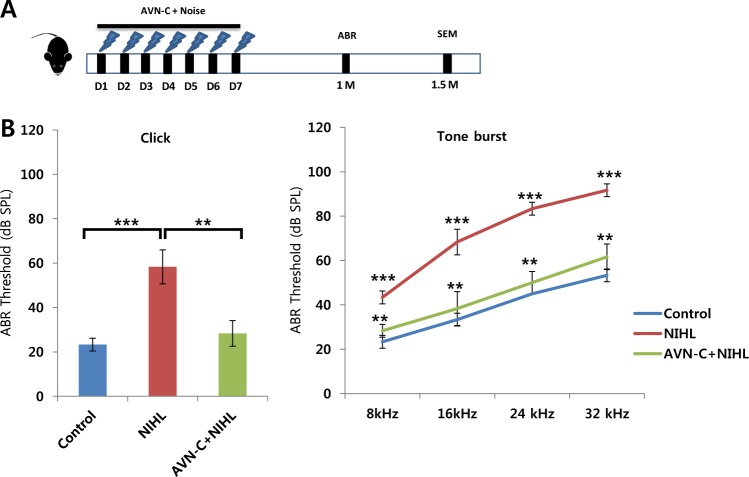
Effect of AVN-C on permanent threshold shift noise-induced hearing loss model. **a** Experimental timeline. A pure tone 8 kHz sound of 105 dB SPL was played for 6 h a day (D) for 7 consecutive days. AVN-C was injected intraperitoneally, every 24 h, 7 times, starting from 30 min prior to the first noise. A month (M) later, the auditory brainstem response (ABR) was evaluated using click and tone burst stimuli. **b** AVN-C prevented permanent hearing loss due to noise trauma. *n* = 3 per each group, *P < 0.05, **P < 0.01, ***P < 0.001

To evaluate whether AVN-C can prevent more severe hearing damage, a permanent threshold shift (PTS) model was used. A pure tone 8 kHz sound of 105 dB SPL was played 6 h a day for 7 consecutive days (*n* = 7 per each group). An IP injection of AVN-C was provided 30 min prior to the noise every day for 7 d. The ABR results, 1 week after the noise cycles, showed an elevated threshold in the noise only group, and AVN-C attenuated this effect (data not shown). A month later, hearing levels were rechecked. The threshold for the noise only group was 58.3 ± 7.6 dB SPL, while that of the AVN-C treated groups was 28.3 ± 5.7 dB SPL on click ABR (Fig. 2, *P* < 0.01). AVN-C successfully prevented permanent HL caused by noise trauma.

### AVN-C crosses the blood-labyrinth barrier, and is detected in the cochlea after systemic injection

**Fig. 3:**
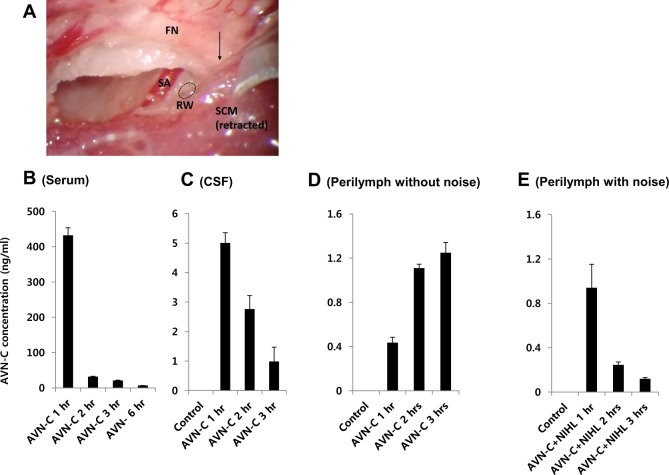
Bioavailability of AVN-C in the cochlea after systemic injection. Overall 10 mg/kg of AVN-C was provided by intraperitoneal injection. Serum, cerebrospinal fluid (CSF), and perilymph were collected at each indicated time point. **a** Harvesting the perilymph through the round window (RW, dotted circle) by a glass pipette (arrow), shown on the left cochlea. **b** AVN-C peaked after 1 h in serum, diminishing thereafter. **c** AVN-C was detected in the CSF at 1 h and gradually decreased. **d** AVN-C was detected in the perilymph after 1 h, increased at 2 h, and was maintained at 3 h. **e** Additional noise during this period lowered the AVN-C levels in the perilymph. FN, facial nerve. SA, stapedial artery. SCM, sternocleidomastoid muscle. *n* = 4 in each group

To determine whether the protection conferred by AVN-C was due to direct effects in the cochlea, we verified the presence of AVN-C in the cochlear fluid after systemic injection. An IP injection of the same dose, of 10 mg/kg of AVN-C, was provided. The AVN-C levels peaked rapidly in the first hour, diminishing thereafter, showing its pharmacodynamics in the blood stream (Fig. 3b). In the case of the cerebrospinal fluid (CSF), the AVN-C level also peaked after 1 h, at a concentration of 5.0 ± 0.3 ng/ml, and then decreased gradually after 2–3 h (Fig. 3c). Perilymph contained AVN-C levels of 0.4 ± 0.04 ng/ml 1 h after systemic injection, revealing that AVN-C penetrates the blood-labyrinth barrier (Fig. 3d). This concentration had increased further after 2 h and was maintained at the 3 h time point. Interestingly, when noise was produced during this period, AVN-C followed the serum and CSF pattern, peaking after 1 h then decreasing (Fig. 3e).

### AVN-C prevents aminoglycoside-induced ototoxicity in vivo

**Fig. 4:**
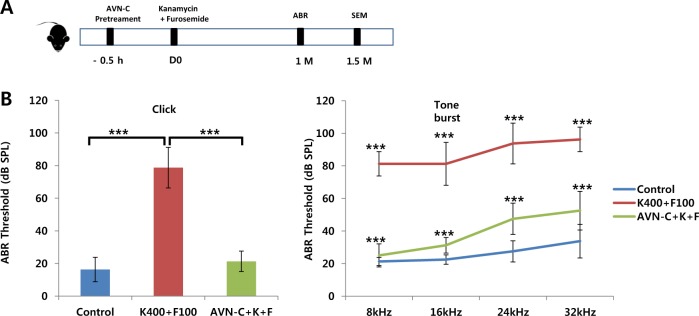
Effect of AVN-C on drug-induced hearing loss due to kanamycin and furosemide (K + F). **a** Experimental timeline. AVN-C was injected intraperitoneally, 30 min prior to kanamycin injection. **b** After 1 month (M), the click auditory brainstem response (ABR) shows that K + F increased the threshold up to 78.75 ± 12.5 dB SPL. AVN-C prevented the threshold shift. Note that the low frequency 8 kHz threshold was also highly elevated by the K + F treatment, while AVN-C rescued it to almost normal. *n* = 3 per each group, *P < 0.05, **P < 0.01, ***P < 0.001

We also investigated the effect of AVN-C on DIHL. For the DIHL model, we used kanamycin, followed by furosemide administration (kanamycin and furosemide (K + F) group). We provided IP AVN-C 30 min prior to the K + F injection. A month later, the hearing threshold of the K + F group was elevated to (78.7 ± 12.5) dB SPL according to click ABR. Compared with noise trauma, K + F affected low frequencies, of 8 kHz (up to 81.3 ± 7.5 dB SPL), as well as higher frequencies in the tone burst ABR. When the AVN-C treatment was used, the click ABR threshold was 21.3 ± 6.2 dB SPL, which is comparable to the normal control. According to the results from the tone burst ABR, each individual frequency threshold was decreased by the AVN-C treatment. The 8 kHz threshold for the AVN-C treated group was almost the same as that of the normal group (Fig. 4).

### Outer hair cells were preserved by AVN-C in noise- and drug-induced hair cell damage

**Fig. 5:**
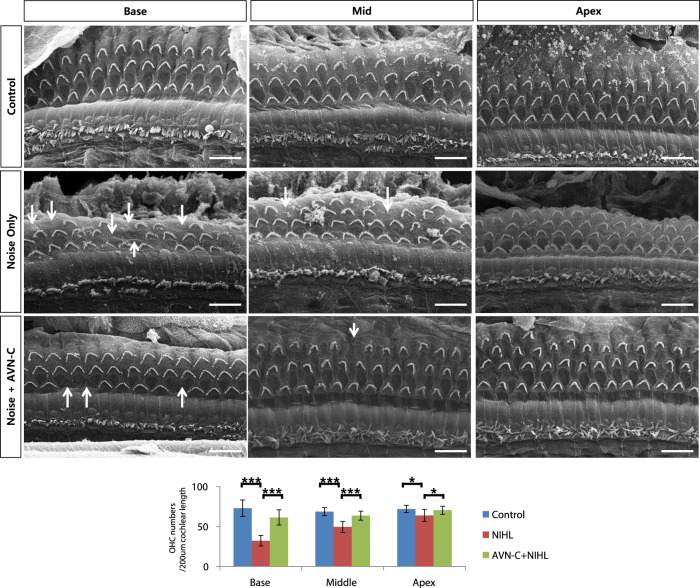
AVN-C reduces outer hair cell (OHC) loss in noise trauma. Scanning electron microscopy images showing missing OHCs (Arrows). AVN-C protects OHCs when used prior to noise. *n* = 3, scale bar: 10 µm. *P < 0.05, **P < 0.01, ***P < 0.001

**Fig. 6:**
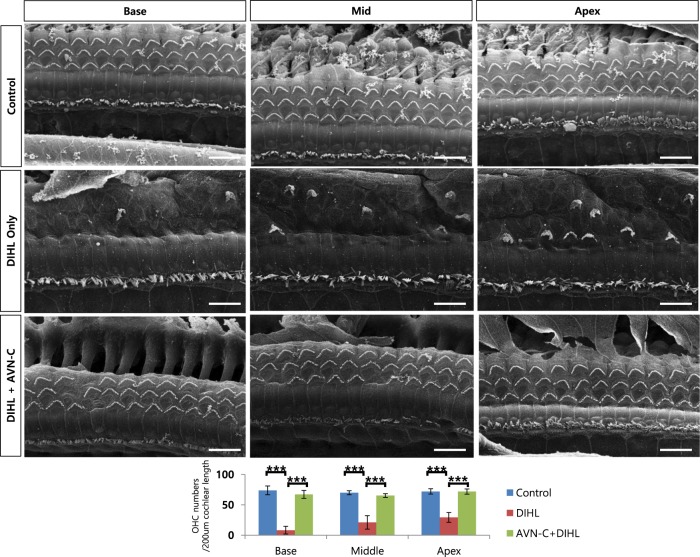
AVN-C strongly prevented outer hair cell (OHC) loss caused by kanamycin and furosemide (K + F). K + F severely damaged OHCs in all cochlear turns. AVN-C protects OHCs when treated prior to K + F. *n* = 5, cochlear middle turn, scale bar: 10 µm. *P < 0.05, **P < 0.01, ***P < 0.001

As hair cells are known to be one of the major components affected by noise or drug ototoxicity, we carried out histological analysis. In the case of the 1-month PTS NIHL, our SEM (Scanning electron microscopy) images show many missing OHCs at the basal and mid turn in the noise only group (control vs noise only group, ((72 ± 10) vs (32 ± 6))/200 μm cochlear length; Fig. 5). The apical cochlear turn was largely preserved. Marked increases in OHC numbers were observed in the case of the AVN-C treatment in the base and mid turn ((61 ± 9) and (63 ± 5)/200 μm *P* < 0.001 compared with noise only group), showing that AVN-C prevented hair cell death. The inner hair cells were all intact, both in the noise only group and the AVN-C-treated group. In the DIHL model with K + F, the OHCs of all cochlear turns were severely damaged (Fig. 6). Strikingly, the AVN-C treatment prevented OHC loss, even in the apical turn. Again, IHCs were preserved in all groups.

### AVN-C reduces ROS production and cytotoxicity in organ of Corti cell line

**Fig. 7:**
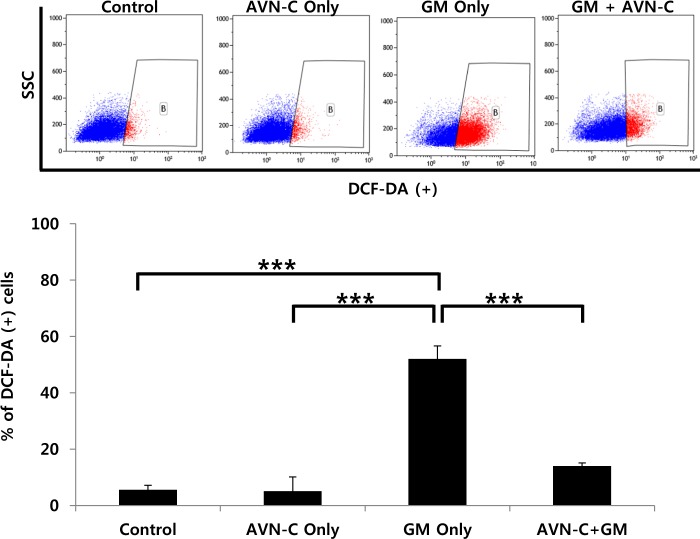
Protective effect of Avenanthramide-C (AVN-C) on gentamicin (GM) induced ROS production in the HEI-OC1 cell line. HEI-OC1 cells were incubated with AVN-C 1 µM alone, GM 3 µM alone, or both, for 24 h. Then, cells were further incubated with dichlorofluorescin diacetate (DCF-DA) for 30 min, harvested, and analyzed by FACS. *P < 0.05, **P < 0.01, ***P < 0.001

As AVN-C is known to be an antioxidant, ROS production was tested using the HEI-OC1 cell line. When 3 μM GM was treated, there were many floating dead cells in the culture media (data not shown), and the fluorescein channel positive population was highly elevated (Fig. 7). Pretreatment with 1 μM AVN-C markedly attenuated the dichlorofluorescin (DCF) positive population, compared with the GM-only treated group ((13.96 ± 1.12) vs (52.02 ± 4.59))%, respectively, *P* < 0.001), showing a reduction in ROS compared with the GM-only treated group. Treating these cell lines with AVN-C yielded similar results to the control.

### AVN-C reduces apoptotic and ROS-related gene expression in both gentamicin-treated HEI-OC1 cells and cochlear tissue with noise trauma

**Fig. 8:**
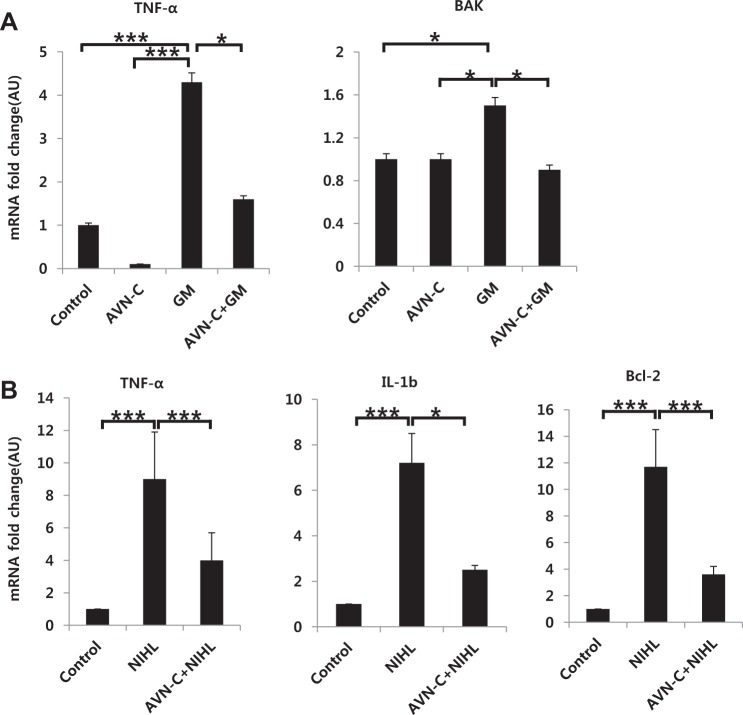
AVN-C reduces reactive oxygen species (ROS) and apoptosis-related gene expression. **a** In vitro HEI-OC1 cell with the GM ototoxicity model. TNF-a and BAK, known to be ROS and apoptosis downstream genes, respectively, were increased in the GM-only group. AVN-C markedly normalized these expressions. **b** Cochlear tissue gene expression after in vivo noise trauma. AVN-C showed a considerable reduction of TNF-α, IL-1b, and Bcl2 genes. *n* = 3. *P < 0.05, ***P < 0.001

To further dissect the downstream pathways modulated by AVN-C, GM-treated HEI-OC1 cells were collected, and mRNA qRT-PCR was performed. TNF-a, which is known to be an important ROS gene, was robustly expressed in the GM-only group. AVN-C again attenuated this expression (Fig. 8a). Furthermore, BAK, an apoptosis-related gene, was highly expressed in the GM-only group, and AVN-C normalized this expression. We also verified this gene expression in the in vivo noise trauma model. Noise trauma elevated TNF-a, IL-1b, and Bcl-2, while AVN-C significantly reduced the expression of these ROS-related genes (Fig. 8b).

## Discussion

Many people are aware of noise trauma, and that it can severely damage hearing. However, the incidence of HL is growing and becoming problematic, especially at younger ages^15^. As there are no treatments other than hearing devices, prevention is the most important approach for addressing SNHL, including NIHL and DIHL. Herein, we showed that AVN-C, a natural compound derived from oats, can serve as a drug for preserving hearing.

Several molecules have been suggested for use as drugs to prevent NIHL. Suberoylanilide hydroxamic acid, a histone deacetylase (HDAC) inhibitor, when IP injected into mice 3 d before noise exposure, preserved hearing and OHCs at the 2-week time point^16^. Similarly, another HDAC inhibitor, sodium butyrate was provided either in drinking water or IP injection to guinea pigs. They were given IP injections once a day for 3 d before and after noise, with further injections 30 min before and 2 h after the noise. They also observed hearing and OHC preservation 2 weeks after noise trauma^17^. However, the HDAC inhibition mechanism is not understood. Though somewhat less effective, Pioglitazone^18^, Atorvastatin^19^, and water-soluble Coenzyme Q10^20^ were reported to reduce NIHL in either rat or guinea pig models. Interestingly, these were all related to modulation of oxidative stress, suggesting that this is the key mechanism of NIHL.

In the case of the prevention of DIHL, many studies have focused on HL caused by cisplatin. Bucillamine^21^, dunnione^7^, R-phenylisopropyladenosine^22^, Forskolin^23^, and alpha-lipoic acid^24^ had been demonstrated by in vivo animal studies to have protective effects against cisplatin ototoxicity. Rescue of aminoglycoside-induced HL has also been reported. Paeoniflorin^25^, dexamethasone, melatonin, and tacrolimus^26^ have prevented the deterioration of hearing due to neomycin and GM, respectively. In DIHL studies including cisplatin and aminoglycoside, all molecules have been shown to ameliorate ROS production. These results indicate that oxidative stress is an effective target for preventing both NIHL and DIHL. Few studies have reported therapeutic effects on both NIHL and DIHL. To the best of our knowledge, there has only been one report, using kenpaullone, a CDK2 kinase inhibitor, relating to protection against HL due to cisplatin and noise^27^. In this paper, we demonstrated the powerful preservative properties of AVN-C in the cases of both NIHL and DIHL due to K + F.

We investigated the effect of AVN on SNHL, as it is a polyphenol, and polyphenols are known to be effective antioxidants^12^. Moreover, as AVN is a natural derivative of oats, it would make an easy to use and safe medication. AVN has been reported to prevent atherosclerosis due to its antiinflammatory effects^13^. The effects of AVN-C on HL are quite astonishing. It rescued both TTS and PTS HL due to noise. The click ABR threshold shift was comparable to the normal control, which was not subjected to any noise trauma. DIHL caused by K + F severely destroyed OHCs, even in the apical turn. AVN-C rescued this damage to nearly normal, as indicated by the ABR and SEM results (Figs. 4 and 6).

In the blood stream, AVN-C quickly reached its peak, and was washed out thereafter (Fig. 3a). This is similar to previous reports in human^28^ and rat^29^ models. They were both given AVN orally, and it still reached its peak after 1–2 h, and then gradually decreased. In our case, we gave mice AVN-C via the IP route, which may have influenced the pharmacodynamics a little faster than an oral preparation. Nonetheless, AVN-C seems to be absorbed and act quickly in the body. As AVN-C is an antioxidant, it may work systemically. We wanted to determine whether the hearing protection conferred by AVN-C was a direct effect, working inside the cochlea. For this, we looked for AVN-C in the perilymph after providing it systemically. AVN-C was detected in the perilymph 1–3 h after systemic injection, indicating that AVN-C penetrates the blood-labyrinth barrier, and can work directly in the cochlea. To the best of our knowledge, this is the first study to confirm the direct presence of a hearing protecting drug in the cochlea.

Compared with its bioavailability in the CSF, which decreased after 1 h, the concentration of AVN-C in the perilymph had increased at 2 h, which demonstrates its cumulative effect after penetrating the blood-labyrinth barrier. This concentration was maintained at 3 h. According to the previous literature, the half-life in the cochlea varies between molecules. For example, dexamethasone-phosphate quickly declines after 2–3 h. Dexamethasone itself is maintained for more than 24 h, and GM was somewhere in between^30^. Moreover, FM 1–43 remains in hair cells for at least 72 h once it has been taken up^31^. We did not check for the presence of AVN-C in the perilymph after 3 h. However, it may be the case that AVN-C can remain in the cochlea for longer periods, maintaining its protective effect. This may explain why it still exhibited protective effects when given 12 or even 24 h before exposure to noise. In any event, there is at least a 24-h time window in which AVN-C contributes to the preservation of hearing in the event of noise trauma.

Interestingly, the concentration of AVN-C reached its peak faster (after 1 h) in the case of noise exposure. The concentration of AVN-C then decreased, following the trends observed in serum and CSF (Fig. 3c, d). This can be attributed to the loosening of the blood-labyrinth-barrier by noise^32^. This suggests that, even though it is sufficiently effective when taken before noise, additional doses with or after noise may support the action of AVN-C. Future study is necessary to optimize the AVN-C treatment schedule for hearing preservation.

The antioxidative role of AVN-C was clearly observed, as expected. DCF is a fluorescence probe that can sensitively detect the total ROS in the cell, and is widely used to measure oxidative stress^33^. When GM was used to treat HEI-OC cells, the DCF-positive population increased significantly. When GM and AVN-C were provided simultaneously, this effect was significantly attenuated. We also analyzed genes known to be activated by oxidative stress. TNF-a and BAK were elevated by GM on HEI-OC cells. AVN-C decreased the expression of these genes. These effects were also confirmed in vivo. When AVN-C was provided before noise, the expressions of TNF-a, IL-1b, and Bcl-2 were all attenuated. AVN-C had definitive antioxidative effects in the cochlea.

## Conclusions

AVN-C has strong preventative effects with respect to hearing deterioration and OHC loss caused by both noise trauma and kanamycin + furosemide. When administrated systemically, AVN-C penetrates the blood-labyrinth barrier, and acts directly on the cochlea. AVN-C reduced the oxidative stress caused by noise and ototoxic drugs. As AVN-C can be provided through dietary products derived from oats, it is a convenient and effective candidate drug for preventing NIHL and DIHL.

## Materials and methods

### Animals and generation of hearing loss models

We used wild type mice of C57Bl/6 background, aged from 3 to 4 weeks. The mice were sheltered in a standard conditioned vivarium, with free access to food and water. The care and use of the animals in this study were approved by the Institutional Animal Care and Use Committee at Chonnam National University Medical School.

For noise exposure, animals were kept in individual cages (12 cm wide), and placed in an electrically shielded, double-walled sound attenuating chamber. We used a sound generator consisting of a Beltone 2000 audiometer (USA) and a power amplifier (93–776 Inter-M R300plus). The noise stimulus was presented through dynamic loudspeakers (JBL 2446J) suspended 10 cm above the animal’s head. To induce the TTS, animals were exposed to a single noise of 95 dB SPL, at 8 kHz pure tone, for 6 h. For a PTS, the same 8 kHz pure tone, at 105 dB, was presented once a day for 7 consecutive days. For the DIHL models, four animals were used for each group, and treated with a single dose of kanamycin 400 mg/Kg by subcutaneous injection, 30 min before furosemide 100 mg/Kg by IP injection. The animals were monitored for 30 d.

### AVN-C administration and detection in tissue fluids

AVN-C was purchased from Sigma-Aldrich (Cat. SI-033-053-1,USA), dissolved in 5% kolliphor or dimethyl sulfoxide (DMSO) for in vivo and in vitro use, respectively. The dose of AVN-C was 10 mg/kg, which was 30 min prior to each noise time course. The same dose of AVN-C was given to drug-treated animals 30 min prior to K + F treatment. The control group only received the carrier, 5% kolliphor.

To analyze the serum, the whole blood was collected by cardiocentesis after anesthesia. The samples were left at room temperature (RT) for 15 min, then centrifuged at 151 × *g* for 5 min. The supernatant serum was gathered and stored at a temperature of −20 °C, before being sent for AVN-C detection in the fluid.

To obtain perilymph, the mice were anesthetized, and their heads fixed. After a skin incision, the subcutaneous fat layer was dissected, with a gentle removal of muscles to reveal the tympanic bulla periosteum. The bulla was encapsulated by gradual removal of bony fragments, until the round window niche was exposed. A glass pipette was penetrated gently through the round window niche, and perilymph was harvested.

To collect CSF, the mice were placed prone on the stereotaxic instrument with direct contact to a heating pad. The head was secured by head adaptors. Incision and careful dissection through postneck muscles revealed the dura mater of the cisterna magna as a glistering and clear reverse triangle, through which the medulla oblongata and a major blood vessel (arteria dorsalis spinalis), and CSF space were visible. The membrane was punctured with a glass pipette, and the fluid was harvested.

Each sample fluid was analyzed by liquid chromatography–mass spectrometry (LC–MS/MS, AB SCIEX 4000 Q Trap mass spectrometer, Shimadzu LC 20A System), to detect AVN-C. The MS condition used Turbo Ion Spray, a temperature of 500 °C, MRM scan type, positive mode, spray voltage 5500 V, CG 20, GS1 50, and GS 60 (AVN-C m/z 316.169/163.000, loperamide m/z 477.223/266.200). The local conditions for the various components were: column: Gemini C18 3.0 µm, (150 mm × 3.0 mm) equipped with a Gemini C18 (4.0 mm × 2.0 mm) guard cartridge, the column oven was at 40 °C, and the auto sampler was at 4 °C. Mobile phase ACN: DIW = 40:60 (V/V) with 0.1% formic acid, and the flow rate was 0.3 ml/min. A standard stock solution of AVN-C used was: 1 mg/ml in DMSO, and loperamide: 1 mg/ml in methanol (Internal standard, IS).

The working standard solutions of AVN-C were 0.5, 1, 2, 5, 20, and 50 ng/ml in methanol and loperamide: 1 ng/ml in 50% methanol (IS) were applied, and the calibration concentrations were B, 0.05, 0.1, 0.2, 0.5, 2, and 5 ng/ml in artificial CSF with a calibration range of 0.05–5 ng/ml.

### Auditory brainstem response for animal hearing evaluation

We recorded the ABR with a 3RZ6 TDT system (Tucker-Davis Technologies, FL, USA), which provided stimuli ranging from clicks to tone bursts. Needle electrodes of length 1.5 mm were inserted subdermally at the dorsal midline between the eyes (none inverting) at the scalp, posterior to both pinnae. At each frequency, we tested various stimuli intensity levels in decreasing order, from 90 to 10 dB of the visual ABR threshold.

### Scanning electron microscopy

Cochleae were rapidly dissected from the cranial bone of the mouse, one animal at a time, to minimize the amount of time between death and fixation (typically 2 min) at RT. Then, 500 µl of fixative, containing 4% paraformaldehyde and 2.5% glutaraldehyde in 0.1 M sodium cacodylate buffer, was gently perfused through the open oval and round window, exiting through the hole made in the apical turn of the cochlea. Tissues were then postfixed overnight at 4 °C on a rotating platform, rinsed three times with distilled water, decalcified in 10% EDTA in 100 mM Tris pH 7.4 for 1 h, and then rinsed twice again. The cochlea turns were dissected, and postfixed in 1% osmium tetroxide for 2 h at RT. The samples were then dehydrated with serial ethanol washings from 50% to absolute ethanol, critical point dried, mounted on support stubs with carbon tabs, and sputter coated with platinum. Imaging was carried out using an EM-30AX PLUS^+^ COXEM (South Korea) scanning electron microscope, operating at 15 kV.

### In vitro HEI-OC1 culture and induction of cytotoxicity

HEI-OC1 cells were cultured under permissive conditions (33 °C). The medium consisted of high-glucose Dulbecco’s modified Eagle’s medium (Gibco BRL, Gaithersburg, MD, USA) containing 10% fetal bovine serum (Gibco BRL) and 50 U/ml gamma interferon (Genzyme, Cambridge, Mass, USA) without antibiotics. AVN-C was dissolved in DMSO, and used in doses of 1 μM. Cells were incubated with GM 3 μM for 24 h, to induce cytotoxicity.

### Flow cytometric analysis

Cell-permeant reagent 2′,7′-dichlorofluorescin diacetate (DCF-DA) was given to the culture media 30 min prior to the collection of HEI-OC1 cells. As DCF-DA is oxidized by ROS and released DCF is a highly fluorescent compound (maximum excitation and emission spectra of 495–529 nm, respectively), the cells were characterized using flow cytometry. BD FACS Calibur (BD FACS Calibur™ flow Cytometer, BD Biosciences, San Jose, CA, 95131, USA) was used. We evaluated the changes in the ROS as a percentage of control after background subtraction using Kaluza Analysis Software (Beckman Coulter, Inc. Brea, CA, 92821, USA).

### RNA isolation and real-time polymerase chain reaction for downstream gene analysis

Either HEI-OC1 cells or cochlear whole tissues were harvested, and total RNA was extracted with Trizol reagent (Invitrogen). The quantity of RNA was determined by spectrophotometry (Spectrophotometer ND-1000 Nano Drop, Technologies Inc., Wilmington, USA) using absorbance at A260/A280 nm, and the results were analyzed using ND-1000 Software. The experiments were carried out three times, and each sample was assayed in triplicate. Denaturation was performed for 10 min and 10 s at 95 °C, and the annealing phase took place at 62 °C for 20 s, followed by 72 °C during 30 s with 40 cycles. The primers for each gene are as follows: GAPDH_For (5′-ACC ACA GTC CAT GCC ATC AC-3′); GAPDH_Rev (5′- TCC ACC ACC CTG TTG CTG TA-3′); IL-1b_For (5′- GAG TGT GGA TCC CAA GCA AT-3′); IL-b_Rev (5′- ACG GAT TCC ATG GTG AAG TC-3′); Bcl-2_For (5′- GTC GCT ACC GTC GTG ACT TC-3′); Bcl-2_Rev (5′- CAG ACA TGC ACC TAC CCA GC-3′); TNFa_For (5′-CCT GGC CTC TCT ACC TTG T-3′); TNFa_Rev (5′- GTC ACC AAA TCA GCG TTA TTA AG-3′); BAK_For (5′-GGT GAA CTG GGG GAG GAT TG-3′); BAK_Rev (5′-AGA GCG ATG TTG TCC ACC AG-3′).

### Statistical analysis

Data were analyzed using SPSS 17.0 *(SPSS Inc., Chicago, IL, USA)*. The independent Student’s *t*-test was used, and a *P* value of less than 0.05 was considered to indicate statistical significance.
